# Monocytes from Uninfected Neonates Born to *Trypanosoma cruzi*-Infected Mothers Display Upregulated Capacity to Produce TNF-α and to Control Infection in Association with Maternally Transferred Antibodies

**DOI:** 10.3390/pathogens12091103

**Published:** 2023-08-29

**Authors:** Amilcar Flores, Cristina Alonso-Vega, Emmanuel Hermann, Mary-Cruz Torrico, Nair Alaide Montaño Villarroel, Faustino Torrico, Yves Carlier, Carine Truyens

**Affiliations:** 1 Facultad de Medicina, Universidad Mayor de San Simon (U.M.S.S.), Cochabamba 2500, Bolivia; 2 Laboratory of Parasitology, Faculty of Medicine, ULB Center for Research in Immunology (UCRI), Université Libre de Bruxelles (U.L.B.), 1070 Brussels, Belgium; 3 Department of Tropical Medicine, School of Public Health and Tropical Medicine, Tulane University, New Orleans, LA 70118, USA

**Keywords:** congenital chagas disease, monocytes, *Trypanosoma cruzi*-specific antibodies

## Abstract

Activated monocytes/macrophages that produce inflammatory cytokines and nitric oxide are crucial for controlling *Trypanosoma cruzi* infection. We previously showed that uninfected newborns from *T. cruzi* infected mothers (M+B- newborns) were sensitized to produce higher levels of inflammatory cytokines than newborns from uninfected mothers (M-B- newborns), suggesting that their monocytes were more activated. Thus, we wondered whether these cells might help limit congenital infection. We investigated this possibility by studying the activation status of M+B- cord blood monocytes and their ability to control *T. cruzi* in vitro infection. We showed that M+B- monocytes have an upregulated capacity to produce the inflammatory cytokine TNF-α and a better ability to control *T. cruzi* infection than M-B- monocytes. Our study also showed that *T. cruzi*-specific Abs transferred from the mother play a dual role by favoring trypomastigote entry into M+B- monocytes and inhibiting intracellular amastigote multiplication. These results support the possibility that some M+B- fetuses may eliminate the parasite transmitted in utero from their mothers, thus being uninfected at birth.

## 1. Introduction

Chagas disease, caused by the protozoan parasite *Trypanosoma cruzi*, is a major cause of morbidity and mortality in Latin America, mainly by inducing a chronic cardiomyopathy [1]. The parasites may be transmitted by the dejections of blood suckling vector bugs, transfusion of infected blood, organ transplantation, or congenitally from the mother to the fetus. National control programs of vectorial and transfusional transmission in various South American countries have reduced the prevalence to around 6–8 million infected people, and the number of exposed individuals to around 60 million [2]. Subsequently, the maternal-fetal transmission of *T. cruzi* became an increasingly important mode of infection, which is susceptible to be repeated to several siblings and from one generation to another [3,4]. Chagas disease has extended to non-endemic countries owing to the migrations of individuals from endemic countries to North America, Europe, Japan, and Australia. In endemic countries, congenital transmission occurs in approximately 4.7% of infected mothers. In Latin America, the number of babies born to infected mothers has been estimated between 158,000 and 214,000 per year, with an incidence of congenital infection of 8668 cases/year. In the United States and in Europe, 60 to 315 and 20 to 184 congenital infections are expected to occur annually through migrations from LA [5].

The morbidity of congenital infections varies from asymptomatic to severe and fatal [6,7]. Except for infected women of reproductive age who are treated with trypanocidal drugs before pregnancy, it is impossible to prevent congenital transmission in pregnant women, as they may not be treated during pregnancy and no biomarkers of transmission are identified. The mechanisms of maternal-fetal transmission are still under study [3,6,8]. The parasite lineage does not seem to be a determinant of congenital transmission, although some haplotypes may be preferentially transmitted [9,10]. However, a high parasite burden associated with a deficient specific immune response during pregnancy are maternal risk factors for in utero transmission [3,6,11]. The familial clustering of congenital cases (transmission repeated in each pregnancy and/or transgenerational) suggests the existence of maternal genetic or epigenetic risk factors of vertical transmission [3,6,12].

Regarding the fetus, his susceptibility to the infection transmitted in utero has not yet been investigated. The role of the fetal innate immune response, the first line of defense against invading pathogens, can be questioned. Activated monocytes/macrophages that produce inflammatory cytokines and nitric oxide are known to play a pivotal role in controlling *T. cruzi* infection [6,13]. Interestingly, we showed that uninfected newborns from *T. cruzi* infected mothers were sensitized to produce higher levels of inflammatory cytokines than newborns from uninfected mothers [14]. This suggests that their monocytes are more activated and raises the hypothesis that these cells might help in limiting/eliminating congenital infection. We investigated this possibility by comparing the ability of cord blood monocytes from neonates born to *T. cruzi* -infected and uninfected mothers to control *T. cruzi* infections in vitro.

## 2. Materials and Methods

### 2.1. Patients and Blood Obtention

Umbilical cord blood samples were collected from vaginal deliveries at the maternity German Urquidi (Universidad Mayor de San Simon (UMSS), Cochabamba, Bolivia) between 2003 and 2007. Cesarean deliveries were not included. Maternal and congenital infections were diagnosed as previously described [7]. Congenital cases were excluded, and cord blood samples were obtained from two groups of healthy newborns: M+B- (uninfected neonates born to *T. cruzi* infected mothers, n = 19) and M-B- (uninfected neonates born to uninfected mothers, n = 18). The scientific/ethics committee of the UMSS approved this study. We obtained informed written consent from the mothers before the blood collection.

### 2.2. Purification of Cord Blood Mononuclear Cells and Monocytes

Cord blood samples were collected either on sodium heparin (10 mL) or sterile blood bags containing sodium citrate (100 mL, for monocyte isolation) (Baxter, Brazil). After centrifugation to preserve plasma, cord blood mononuclear cells (CBMCs) were isolated by density gradient centrifugation on Nycoprep (Nycomed Pharma AS, Oslo, Norway). The monocytes were purified by adherence. Therefore, 80 to 100 million CBMCs in 15 mL RPMI containing 5% of decomplemented fetal calf serum, 1% L-glutamine, 100 U/mL penicillin G, and 100 µg/mL streptomycin (all from BioWhittaker Europe, Verviers, Belgium), were cultured in 75 cm^2^ flasks pre-coated with heat-inactivated fetal calf serum overnight at 37° in 5% CO_2_ atmosphere. Non-adherent cells were eliminated by discarding the culture medium and adherent cells, corresponding to monocytes, were washed twice with RPMI. After further incubation in cold PBS for 30 min, monocytes were detached using a cell scraper, washed, counted, resuspended in culture medium, and immediately used for the studies. The yield of CMBCs was similar in both groups of neonates (1.32 ± 0.16 and 1.40 ± 0.13 × 10^6^ CBMCs/mL blood from M-B- and M+B- samples respectively, n = 18 and 19, *p* = 0.257, Mann–Whitney test), as were the proportions of adherent cells collected per million CBMCs (M-B- samples; were 2.55 ± 0.28, M+B- samples, 2.05 ± 0.28, n = 19, *p* = 0.093).

### 2.3. T. cruzi Infection of Monocytes

*T. cruzi* trypomastigotes (Tulahuen strain, genotype TcVI [15]) were collected from the supernatant of infected fibroblasts, as previously described [14]. The samples were cryopreserved in liquid nitrogen, defrosted, and washed immediately before use. Monocytes were cultured in microwells of 16 wells glass slides (Labtek, Nunc, Roskilde, Denmark) at 10^5^ monocytes/well in 0.2 mL, of RPMI containing 1% L-glutamine, 100 U/mL penicillin G, and 100 µg/mL streptomycin (all from BioWhittaker Europe, Verviers, Belgium), and either 10% of decomplemented human adult AB+ plasma (Fisher BioReagents, Thermo-Fisher Scientific, Brussels, Belgium) or decomplemented autologous cord plasma. Monocytes were infected with trypomastigotes at different ratios depending on the experiments (parasite to cell ratios of 1:1, 1:5, 1:20 or 1:100, indicated in the figures). Each experiment was performed in duplicates. After different incubation times, the culture medium was discarded, and the cells were washed with PBS, fixed, and stained with Giemsa for microscopic examination of the proportion of infected cells (A) and the mean number of amastigotes per infected cell (B). The parasitic index corresponding to (A × B)/100 was used to evaluate the global parasite burden.

### 2.4. TNF-α Production by Monocytes

Freshly purified monocytes were cultured for 24 h at a concentration of 10^6^ cells/mL in culture medium containing 1% L-glutamine, 100 U/mL penicillin G, and 100 µg/mL streptomycin and 10% fetal calf serum (all from BioWhittaker Europe, Verviers, Belgium) and stimulated with a *T. cruzi* trypomastigote lysate (prepared as described in [14]), used at 10^6^ equivalent parasite/mL, i.e., a ratio of 1 parasite/cell) or lipopolysaccharide (LPS, 0.01 µg/mL, Sigma-Aldrich, Saint-Louis, MO, USA). TNF-*a* levels in the culture supernatants were determined by ELISA using antibody (Ab) pairs from Invitrogen (Thermo Fisher Scientific, Merelbeke, Belgium). Assays were performed in duplicate, according to the manufacturer’s instructions. The detection limit was 2 pg/mL. In other experiments, freshly isolated monocytes were cryopreserved as described elsewhere [16] and sent to Brussels in liquid nitrogen gaseous phase using the transport vessel “Voyageur” (VWR, Leuven, Belgium). After thawing and washings, monocytes were cultured for 4 h with the trypomastigote lysate at a parasite to cell ratio of 1 in the presence of brefeldin (10 µg/mL, Sigma-Aldrich) and intracellular TNF-*a* was measured by flow cytometry using anti-CD14-Percp and anti-TNF-*a*-PE mAbs (from BD Biosciences Benelux, Erembodegem, Belgium). Data were acquired on a BD FACSCalibur flow cytometer and analyzed using the BD Cell Quest software (version 6.0).

### 2.5. Phenotypic Characterization of Monocytes by Flow Cytometry

CBMCs isolated from M-B- and M+B- cord blood samples were cryopreserved and sent to Brussels for flow cytometry analysis. The cells were thawed, washed, and stained as previously described [11]. The following mAbs and their matched control isotypes were used together: either anti CD14-Percp, anti-HLA-DR-FITC, and anti-CD54-PE, or anti CD14-Percp and anti-TLR2-PE, or anti CD14-Percp and anti-TLR4-PE (all from BD Biosciences). Data were acquired using a BD FACSCalibur flow cytometer. We then gated the CD14^+^ monocytes and studied the mean fluorescence intensity (MFI) of the markers and the proportion of positive cells using the BD Cell Quest software (version 6.0).

### 2.6. Depletion of T. cruzi-Specific Antibodies by Affinity Chromatography

Immunosorbent was prepared by coupling 7 mg of a soluble antigenic extract of cultured *T. cruzi* trypomastigotes, as described elsewhere [17] to 1 g of CNBr-activated Sepharose^®^ 4 B (Amersham Biosciences, Buckinghamshire, UK) following the manufacturer’s instructions. The Sepharose was saturated with glycine. A mock immunosorbent assay was performed using glycine alone. To deplete *T. cruzi*-specific antibodies from cord blood plasma, we incubated 100 µL of plasma sample with 100 µL of immunosorbent for 1 h at room temperature. After sedimentation of Sepharose, the supernatant was recovered and sterilized through a 0.22 µm membrane. *T. cruzi*-specific antibodies were quantified by ELISA before and after immunoadsorption using soluble antigenic *T. cruzi* trypomastigote extract and peroxidase-labelled goat antibodies recognizing human IgG, A, and M (H+L) (Bio-Rad Laboratories, Steenvoorde, France).

## 3. Results

### 3.1. Monocytes from M+B- Newborns Are Primed to Produce Higher TNF-α Levels

**Figure 1:**
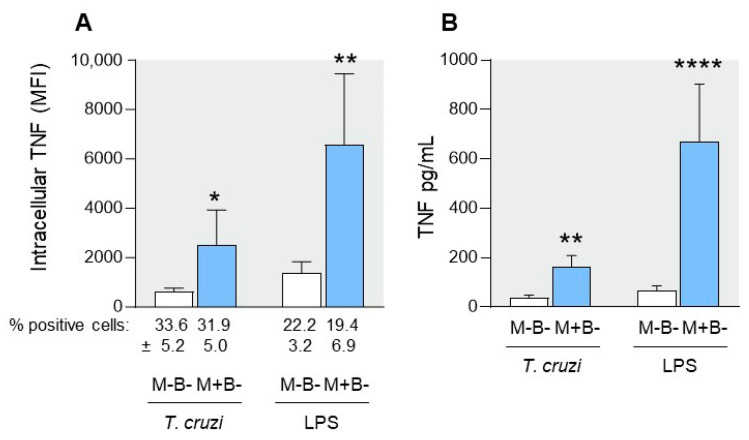
TNF-*a* expression by monocytes from M+B- and M-B-newborns. (**A**) Purified cord blood monocytes were cultured for 4 h with a *T. cruzi* trypomastigote lysate (10^6^ parasites/mL, parasite to cell ratio of 1) or LPS (0.01 µg/mL), in the presence of brefeldin (10 µg/mL). The proportion of TNF-*a*-positive CD14+ cells and intracellular TNF-*a* levels (shown as mean fluorescence intensities—MFI) were evaluated by flow cytometry. Results are expressed as mean ± SEM of 9 M-B- and 11 M+B- samples. (**B**) In other similar experiments in which brefeldin was not added, TNF-*a* was measured in supernatants by ELISA. Results are expressed as mean ± SEM (n = 11 M-B- samples and 14 M+B- samples). *: *p* < 0.05, **: *p* < 0.005, ****: *p*< 0.0001 vs. M-B- group (Mann–Whitney test).

We evaluated the activation state of cord blood CD14+ monocytes from M+B- and M-B- newborns by studying their surface expression of CD54 and HLA-DR and their capacity to produce TNF-α. Although M+B- and M-B-cord blood monocytes expressed similar levels of CD54 and HLA-DR (Table 1 and Supplementary Figure S1), M+B- monocytes expressed and released significantly 4–10 times more TNF-α in the supernatant when cultured in the presence of a lysate of *T. cruzi* trypomastigotes or LPS, although the proportion of monocytes expressing TNF-*a* did not vary between newborn groups (Figure 1). This increased TNF-α production was not related to differences in the expression levels of TLR2 and TLR4 (Table 1 and Supplementary Figure S1), which are known to be engaged by *T. cruzi* (both TLRs) and LPS (TLR4) and trigger the production of inflammatory cytokines [17,18]. These results indicate that monocytes from M+B- newborns are primed to produce more TNF-α than M-B-monocytes.

### 3.2. Susceptibility of M+B- Monocytes to T. cruzi Infection

**Figure 2:**
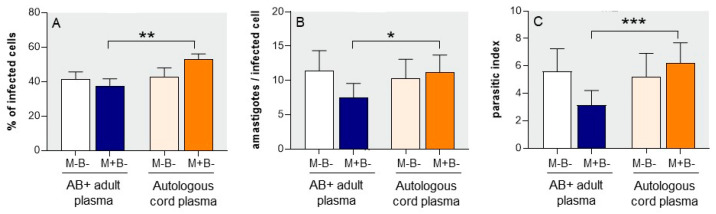
Initial parasite burden of monocytes from M+B- and M-B-newborns, infected with *T. cruzi* trypomastigotes (ratio 1 parasite/cell) for 22 h, in the presence of AB+ adult human or autologous cord plasma. The proportion of infected monocytes (**A**) and the number of amastigotes per infected cell (**B**) were determined by microscopic examination. The parasitic index (**C**) evaluates the global parasite burden (see M&M). Results are expressed as mean ± SEM (n = 10 in each group). *: *p* < 0.05, **: *p* < 0.05, ***: *p* < 0.005 as compared to M+B- monocytes cultures with AB+ adult plasma (Mann–Whitney–Wilcoxon test).

We compared the susceptibility to *T. cruzi* infection of monocytes from M+B- and M-B-newborns, cultured with a ratio of 1 trypomastigote per cell for 22 h. Trypomastigotes are known to rapidly invade cells and escape into the cytoplasm as amastigotes. As the latter remain quiescent for 24 to 35 h before initiating their replication [19,20], we assumed that the parasite burden at 22 h mainly reflected the entrance of parasites into cells. Cultures were performed in the presence of either AB+ plasma from adult donors or autologous cord plasma. Figure 2A shows that at 22 h, in the presence of AB+ adult plasma, a similar proportion of M-B- and M+B- monocytes was infected (41.4 ± 4.2 and 37.7 ± 4.0%, respectively). At this time point (22 h), M-B- infected monocytes contained meanly 11.4 ± 2.9 amastigotes/cell (Figure 2B) while M+B- monocytes contained less amastigotes (7.5 ± 2.8 parasites/cell), resulting in a lower global parasite burden illustrated by the parasitic index (Figure 2C). The use of autologous cord plasma instead of adult AB+ plasma did not modify the parasite burden of M-B-monocytes, either in terms of the proportion of infected cells or the mean number of intracellular parasites at 22 h (Figure 2). However, autologous M+B- cord plasma increased the parasite burden of M+B- monocytes, which was nearly doubled as compared to the use of AB+ adult plasma (index of 6.2 ± 1.5 vs. 3.2 ± 1.1—Figure 2C), resulting from rises of both the proportion of infected cells (52.8 ± 3.3 vs. 37.6 ± 4.0%) and the mean number of intracellular parasites (11.2 ± 2.6 vs. 7.5 ± 2.1).

These results highlight two peculiarities of M+B- newborns as compared to the M-B- ones: their monocytes seem to be inherently less permissive to *T. cruzi* entry of trypomastigotes (cf. when cultured with AB+ adult plasma), while M+B- autologous cord plasma promoted *T. cruzi* infection of M+B- monocytes.

### 3.3. Autologous Cord Plasma Limits Intracellular T. cruzi Multiplication

**Figure 3:**
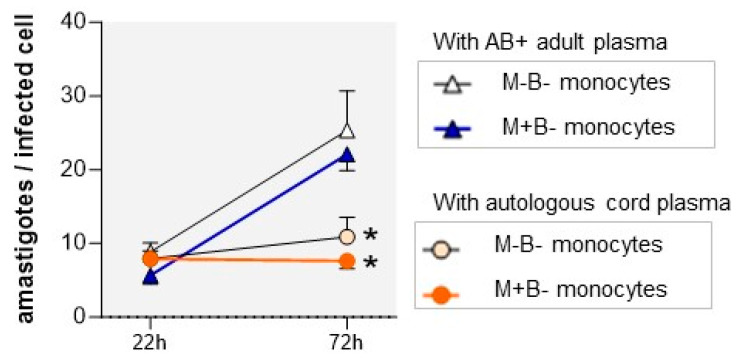
Intracellular amastigote multiplication in monocytes from M+B- and M-B-newborns, infected in vitro with *T. cruzi* trypomastigotes (ratio 1 parasite/cell) in the presence of AB+ adult human plasma or autologous cord plasma. The number of amastigotes per infected cell was determined by microscopic examination at 22 and 72 h. Results are expressed as mean ± SEM (n= between 6 and 12). *: *p* < 0.05, as compared to monocytes cultured with AB+ adult plasma (Mann–Whitney–Wilcoxon test).

We next evaluated the ability of M-B- and M+B- monocytes to support intracellular *T. cruzi* multiplication by measuring the evolution of intracellular amastigote counts between 22 h (i.e., after their quiescent period, see above) and 72 h, when parasites are still not released from the cells. Monocytes of both newborn groups cultured in the presence of AB+ adult plasma allowed the amastigotes to multiply at a similar rate (Figure 3). In contrast, the mean number of amastigotes did not increase and remained roughly stable in the monocytes of both newborn groups when cultures were performed in the presence of autologous cord plasma. This suggests that the cord plasma of M-B- and M+B- neonates contains some factor(s) that inhibit intracellular *T. cruzi* multiplication in monocytes.

### 3.4. M+B- Monocytes Are Occasionally Able to Completely Control Intracellular Infection

In another set of experiments, we choose to decrease the parasite-to-cell ratio to 1:5, 1:20, and 1:100. Even if a parasite-to-cell ratio of 1:100 might not accurately represent the in vivo scenario, it probably becomes closer to the in vivo conditions, that is, in case of transplacental passage of few parasites, or better like the ratio between monocytes and parasites encountered in the blood of congenitally infected newborns [3,6]. We also increased the incubation time to 96 h, assuming that a lower parasite burden and a longer incubation time might increase the opportunity for cells to better control the infection. Cultures were performed in the presence of autologous cord plasma.

**Figure 4:**
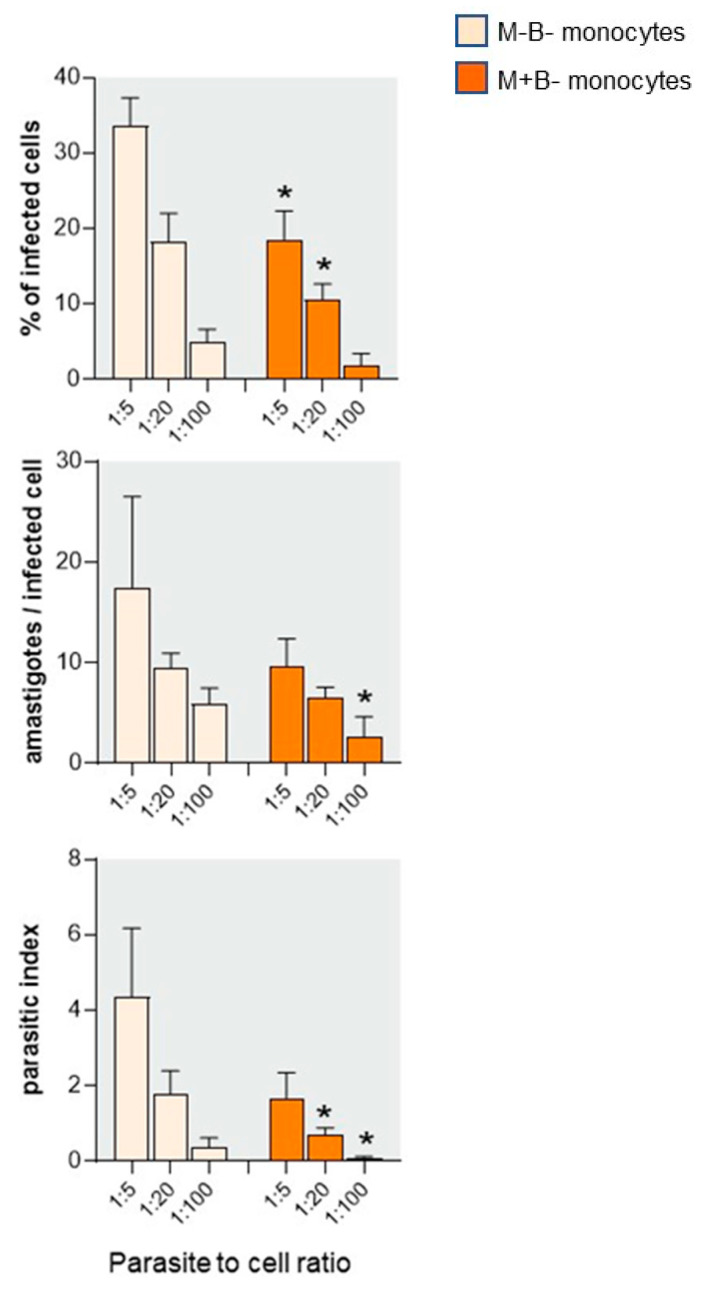
Parasite burden of monocytes from M-B- and M+B- cord blood infected with low doses of *T. cruzi* trypomastigotes and cultured during 96 h in the presence of autologous cord plasma. After 96 h, cultures were stopped, cells were colored, and we counted the number of infected cells and of intracellular amastigotes per infected cell. The index corresponded to the product of both parameters and evaluates the global parasite burden. Results are expressed as mean ± SEM (n = 5). *: *p* < 0.05 compared with M-B- monocytes cultured with the same parasite ratio (Mann–Whitney–Wilcoxon test).

Figure 4 shows that the proportion of infected cells and the number of intracellular amastigotes expectedly decreased when lower parasite to monocyte ratios were used, in monocytes from both newborn groups. These parameters were also consistently lower in M+B- monocytes than in M-B- monocytes by approximately two to three times. More interestingly, with the lowest parasite to cell ratio (1:100), at 96 h the parasite burden was even six times lower in M+B- monocytes than in M-B- monocytes (parasitic index of 0.07 ± 0.03 vs. 0.39 ± 0.24), and no amastigotes were observed in the monocytes of one of the five newborns tested. Altogether, these results confirm that M+B- neonatal monocytes are less susceptible to *T. cruzi* infection and have a better ability to control infection when they encounter low parasite amounts.

### 3.5. T. cruzi–Specific Antibodies Present in the Plasma of M+B- Newborns Improve the Control of the Infection

**Figure 5:**
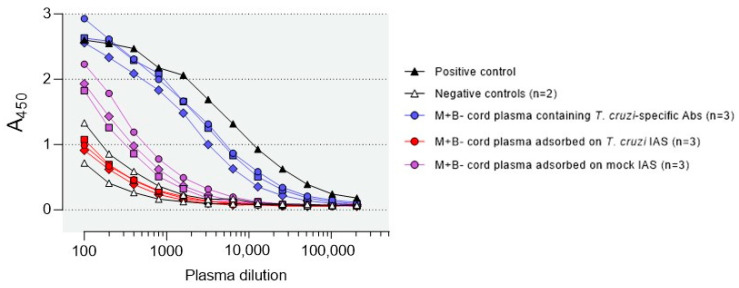
*T. cruzi*-specific Ab in the plasma of M+B- newborns before and after Ab depletion by immunoadsorption on adsorbent containing a *T. cruzi* antigenic extract or a mock IAS (Sepharose saturated with glycine). Ab were measured by ELISA. Sera from adults infected or not with *T. cruzi* were used as positive and negative controls.

As M+B- cord plasma contains *T. cruzi*-specific IgG transferred from the infected mother, we wondered whether they might play a role in the control of in vitro infection by neonatal monocytes. To study this, we compared the infection levels in M+B- monocytes incubated with autologous M+B- plasma containing or not *T. cruzi*-specific antibodies. Abs were depleted by immunoadsorption on *T. cruzi* antigenic extract linked to Sepharose. As a control for the plasma treatment, another aliquot of the plasma sample was adsorbed on the mock immunosorbent assay. Antibody depletion was verified by ELISA. Figure 5 shows that they were completely depleted when Sepharose was coupled with *T. cruzi* antigenic extract, whereas non-depleted M+B- newborn plasma contained Ab levels similar to those of an adult patient chronically infected with *T. cruzi*. Plasma samples that were incubated with the mock immunosorbent assay showed reduced levels of *T. cruzi*-specific Ab. This is probably due to the elimination of Abs recognizing galactosyl residues of Sepharose^®^ 4B (a resin composed of agarose beads and a polymer of Gal β1,4 [3,6]-anhydro-L-galactose), since the production of anti-GAL Abs is strongly stimulated in *T. cruzi* infection [21,22].

**Figure 6:**
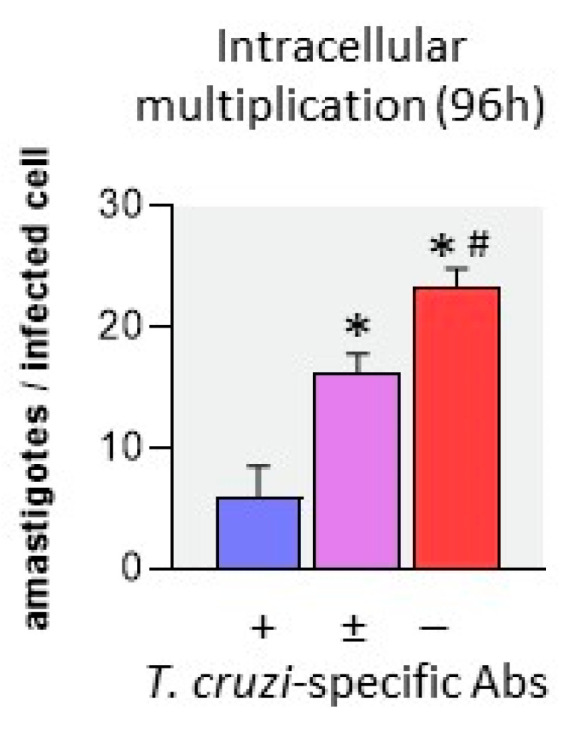
Intracellular amastigotes in monocytes from M+B- newborns after 96 h of infection. Monocytes were infected with *T. cruzi* trypomastigotes at a ratio of 1 parasite/20 cell in the presence of autologous M+B- cord plasma depleted or not from *T. cruzi*-specific Abs: X axis: “+”: Abs are present (undepleted plasma); “±”: part of Abs are depleted (cf. Figure 5); “−“Abs are totally depleted. The number of amastigotes per infected cell was determined by microscopic examination. Results are expressed as mean ± SEM (n = 5). *: *p* < 0.05, as compared to the presence of Abs, #: *p* < 0.05 as compared to half-depleted Abs (Mann–Whitney–Wilcoxon test).

Figure 6 shows that, after 96 h of culture, the lower the level of *T. cruzi*-specific Ab were, the higher were the number of intracellular amastigotes. This suggests that specific Abs limit intracellular multiplication of *T. cruzi* amastigotes in M+B- monocytes. Thus, parasite burden was significantly lower in the presence than in the absence of a specific Abs.

## 4. Discussion

This study identified two traits of M+B- as compared to M-B- neonates: their upregulated capacity to produce the inflammatory cytokine TNF-α and their inherent better ability to control *T. cruzi* infection. Our study also showed that *T. cruzi*-specific Abs transferred from the mother play a dual role by promoting trypomastigote invasion into monocytes and inhibiting intracellular amastigote multiplication.

Our previous study revealed the higher ability of M+B- cord blood cells to produce inflammatory cytokines upon in vitro stimulation by a *T. cruzi* lysate or LPS compared to M-B- cells [14]. Our present results confirm this observation, identifying monocytes as a source of upregulated TNF-α. The phenomenon of in utero sensitization of neonatal cells to produce inflammatory cytokines by chronic maternal infection in the absence of congenital infection has since been shown in cases of various chronic maternal infections such as HIV, HBV, HCV, and malaria (reviewed in [23]). The paradigm of trained immunity, that is, the phenomenon of augmented innate immune function following a stimulus that is not specific to the original stimulus, was proposed in 2011 by Netea et al. [24]. Trained immunity is mediated by extensive metabolic and epigenetic reprogramming of cells of the innate immune system [25]. Trained immunity has been reported to likely accounts for the effect of maternal infections on the fetal/newborn innate immune system [26]. In line with this, we showed that chronic maternal *T. cruzi* infection induced modifications of immune responses to post-natal vaccinations in infants [27] and more recently that it modified the DNA methylation profile of cord blood cells of uninfected newborns [28].

How and which factors are transmitted from the *T. cruzi*-infected mother to her fetus to impact the immune system remains unclear. Chorioamnionitis, sometimes encountered in the placenta of uninfected fetuses from *T. cruzi*-infected mothers [3,29], has been reported as a potential source of sensitizing stimuli [30]. Transplacental transfer of sensitizing *T. cruzi* components from the infected mother to her fetus is another possibility [26], although molecules implicated have not yet been identified. As TNF-a expression is critically induced by histone methylation triggered by the TLR4 ligand LPS [31] and as *T. cruzi* expresses TLR4 ligands [32,33], we may wonder if such molecules might be transferred from the infected mother to prime fetal monocytes. We cannot discard the potential effect of IgG and/or *T. cruzi*-specific Abs transferred from the mother. Indeed, terminally sialylated IgGs have been shown to exert anti-inflammatory effects [34,35]. *T. cruzi* possesses a unique enzyme, trans-sialidase (TS), which cleaves terminal sialic acid residues from host donor glycoconjugates and transfers them onto parasite surface mucins [36,37]. Using this enzyme, *T. cruzi* may reduce IgG sialylation, thereby favoring a pro-inflammatory priming effect of IgG transferred in utero, as it occurs in various other infections [38]. Whatever the underlying mechanism, the most interesting point is that maternal *T. cruzi* infection sensitizes fetal monocytes to produce TNF-a, known to be involved in the control of *T. cruzi* infection [18,39].

Therefore, we investigated the ability of M+B- neonatal monocytes to control infection, in the presence of either adult or autologous cord plasma, whose compositions differ [40]). In addition, *T. cruzi*-specific IgG are present in M+B- cord plasma and obviously not in M-B- plasma. Regarding the first phase of invasion (<24–35 h post-infection, comprising trypomastigote entry, escape of amastigotes from the phagolysosomes into the cytoplasm and a latency phase before amastigotes begin to multiply [20,41]), a similar proportion of M-B- and M+B+ monocytes was permissive to parasite entry when cultured in adult plasma, though M+B- cells contained fewer amastigotes after 22 h. *T. cruzi* cell invasion and its escape into the cytoplasm is a highly complex process (recently reviewed in [42] and [43]). A hypothesis might rely on the differential expression, by inflammatory M+B- monocytes compared to M-B- monocytes, of receptors mediating the entry of trypomastigotes, such as the fibronectin receptor and the prokineticin receptor PK2R [44,45,46,47]. Another possibility is that, in relation to their activated state, they produce more, or more rapidly, trypanocidal compounds such as nitric oxide and peroxynitrite that kill trypomastigotes in the phagolysosome before they escape into the cytoplasm [48].

The use of autologous M+B- cord plasma instead of adult AB+ plasma resulted in a higher parasite burden in M+B- monocytes at 22 h. Such an increase was not observed in M-B-monocytes cultured with M-B-cord plasma. This suggests that something specifically present in cord M+B- plasma thwarts, compensates or prevents the predisposition of M+B- monocytes to host fewer amastigotes. The most evident hypothesis relies on *T. cruzi*-specific IgGs from the infected mother (absent in non-autologous adult plasma and M-B-cord plasma). By opsonizing parasites, they might favor monocyte invasion (as reviewed in [8]). Plasma levels of adenosine, known to be higher in cord blood than in adult blood [49] might also be increased in M+B- cord plasma in relation to placental inflammation [3,29,50]. Adenosine can inhibit NO production [49,51,52,53]. Accordingly, a higher adenosine amount might limit the early ability of M+B- monocytes to kill trypomastigotes before they escape into the cytoplasm, thereby increasing the number of cytoplasmic amastigotes at 22 h. In addition, other anti-inflammatory factors, such as cytokines produced by regulatory T cells and erythroid cells, are present in the cord plasma and not or at lower concentrations in adults [54,55]. In summary, the inherently lower susceptibility of M+B- monocytes to the first invasion phase by *T. cruzi* (cf. culture with adult plasma) seems to be compensated for by cord blood factors.

Regarding the intracellular replication phase, amastigotes markedly multiplied in the monocytes of both groups cultured in the presence of adult AB+ plasma. In contrast, cord plasma dramatically curbed amastigote multiplication in both groups, suggesting that some plasma components, common to M-B- and M+B- cord plasma, promote intracellular killing by monocytes. It is possible that growth factors (GF), such as VEGF, EGF, and FGF, are present at higher levels in cord plasma than in adult plasma [40]. GFs promote monocyte viability by activating the AKT pathway. Interestingly, the AKT pathway has been identified as a critical checkpoint that regulates the replication of *T. cruzi* amastigotes without affecting pre-replication events [20]. Alternatively, instead of being killed, amastigotes may have entered a quiescent stage, whose existence has recently been disclosed [56].

Amastigote growth inhibition by autologous cord plasma was slightly more pronounced in M+B- monocytes at 72 h. By decreasing hundred times the parasite to cell ratio from 1:1 to 1:100 and extending the culture till 96 h, we could observe a significant difference between M+B- and M-B- monocytes. At a ratio of 1 parasite/100 monocytes, M+B- monocytes better controlled the infection and were even occasionally able to completely clear the parasites. The improved ability of M+B- monocytes cultured in M+B- autologous cord plasma to control infection may result from epigenetic reprogramming absent in M-B- monocytes. Indeed, a recent study in mice showed that “trained” neonatal monocytes not only produced higher levels of inflammatory cytokines but also displayed markedly stronger microbicidal activity against intracellular bacteria [57]. The role of *T. cruzi*-specific IgGs present in M+B- cord plasma has also been considered. Depleting *T. cruzi*-specific Abs from M+B-cord blood resulted in a higher intracellular infection level. The protective effect of IgG Abs might result from diverse FcR-mediated effects, such as higher NO production by the already primed M+B- monocytes following FcγR engagement [58], an increase in the ratio between activating and inhibitory receptors on primed M+B- monocytes, which reduces the activation threshold via FcRs [59], and/or an intracellular effect of IgG internalized via FcRn expressed by monocytes, a mechanism recently proposed for some viruses [60]. Regardless of the mechanism, our results show that maternally transmitted Abs help monocytes to control intracellular infection, even if they favor parasite invasion at the initial step of cell infection, as explained above. It might even be speculated that the association of both phenomena contribute to eliminate still more parasites.

Our study raises the hypothesis that fetal blood monocytes primed by maternal infection, in association with maternally transferred Abs, might contribute to limit the occurrence of *T. cruzi* congenital infection in case of transplacental transfer of few parasites. Another recent study also proposed that maternally transmitted Abs may protect against congenital CMV infection [61]. We previously showed that circulating cord blood monocytes from congenitally infected newborns were less activated than the M+B- ones [11]. We may hypothesize that their ability to fight the infection may become overwhelmed in case of transplacental transmission of a higher number of parasites, in relation to a higher parasite load present in transmitting mothers [3,11]. In other words, it can be argued that some M+B- neonates proceed from abortive in utero infection.

Little is known about the role of fetal placental phagocytes in protection against congenital infection. Hofbauer cells (HBC) are fetal macrophages residing in the stroma of chorionic villi (some are located close to the fetal capillaries) and the chorioamniotic membranes [62,63]. As they express diverse TLRs and display microbicidal activity when exposed to inflammatory stimuli, they may play a critical role for fetal protection against vertical transmission of pathogens [64,65]. We may thus wonder whether HBC would also be primed, similarly to monocytes circulating in neonates, thereby also contributing to protect some fetuses from congenital infection. A protective role of Hofbauer cells has been suggested in other models of infections [66,67].

Our hypothesis that some neonates may proceed from abortive in utero infection is based on a limited sample size, using one parasite strain with samples from a restricted country. It is difficult to know if it may be transposed to in vivo conditions. Nevertheless, the present results bring about a new standpoint i.e., that transplacental transmission of pathogens, whether of *T. cruzi* or others, might not mandatorily result into a congenital infectious disease. Moreover, we may wonder if such a control could also occur after birth during the first days of life.

All of this has practical implications in terms of laboratory diagnosis, treatment and strategies to control congenital infections: (i) parasite detection at birth should be confirmed with a second blood sample later after birth; (ii) laboratory diagnosis should detect living parasites in considering the diagnosis of congenital infection (caution with molecular tests mainly detecting pathogen DNA whether dead or alive [3,5]), (iii) neonate etiological treatment, a long and delicate procedure for the family of a newborn displaying a congenital Chagas disease [5], should be initiated after diagnosis confirmation; and (iv) a strengthening of monocyte activation could be considered as a complementary treatment for a congenital infection.

## Figures and Tables

**Table 1:** Characterization of monocytes in cord blood from M+B- and M-B-newborns.

		M-B-	M+B-
CD54	% (a)	67.1 ± 6.2 (10)	56.1 ± 8.0 (16)
	MFI (b)	14.1 ± 1.1	12.2 ± 2.2
HLA-DR	% (a)	98.0 ± 0.3 (10)	90.6 ± 4.9 (16)
	MFI (b)	95.9 ± 6.8	86.2 ± 12.4
TLR2	% (a)	98.1 ± 0.5 (9)	96.2 ± 1.5 (11)
	MFI (b)	58.8 ± 0.5	52.3 ± 4.4
TLR4	% (a)	76.0 ± 4.8 (9)	75.6 ± 5.1 (11)
	MFI (b)	30.8 ± 1.5	28.6 ± 2.0

(a) proportion amongst CD14+ cells; mean ± SEM (n). (b) mean fluorescence intensity; mean ± SEM [n is the same than in (a)].

## Data Availability

Data are included within the article. The datasets used during the current study are available from the corresponding author upon request.

## Supplementary Materials

The following supporting information can be downloaded at: https://www.mdpi.com/article/10.3390/pathogens12091103/s1, Figure S1: Phenotypic characterization of cord blood monocytes.
