# Supplementary material for: Monocytes from Uninfected Neonates Born to Trypanosoma cruzi-Infected Mothers Display Upregulated Capacity to Produce TNF-α and to Control Infection in Association with Maternally Transferred Antibodies
Source: Pathogens. 2023 Aug 29;12(9):1103. doi: 10.3390/pathogens12091103 (PMC10536721; doi:10.3390/pathogens12091103)
Supplement: Supplementary file 1 [file pathogens-12-01103-s001.zip › pathogens-2561870-supplementary.pdf]

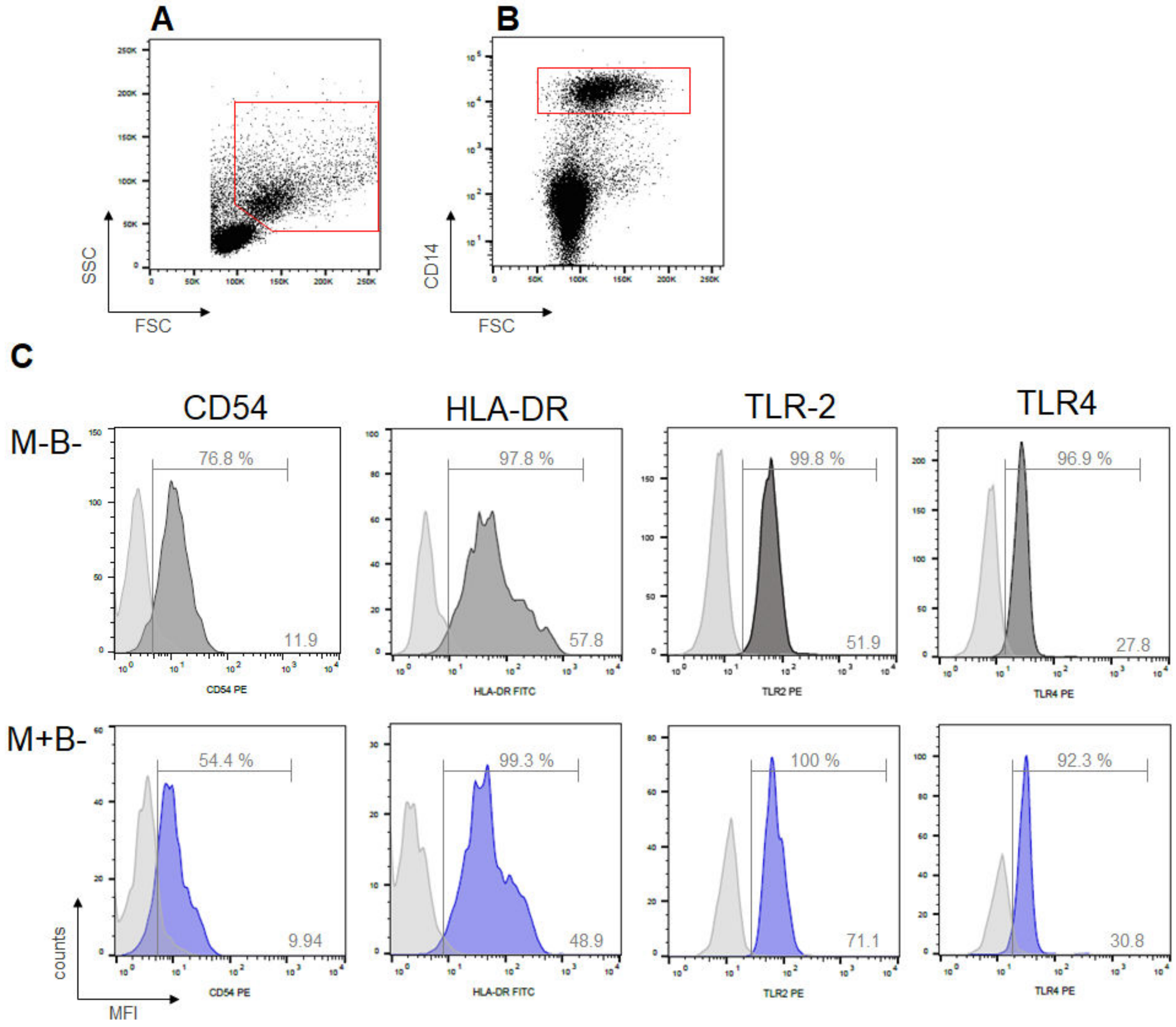

Supplementary Figure S1. Phenotypic characterization of cord blood monocytes. The gating strategy consisted of determining a first gate based on the morphology on cells on the FSC vs. SSC dot plot, which excluded lymphoid cells (**A**), followed by a gate on cells highly expressing CD14 (**B**). The studied marker was then analyzed on CD14+ cells. (**C**) shows an example of results obtained with one M-B- and one M+B- cord blood samples. Light gray histograms correspond to the control isotype, dark grey and blue histograms show the marked cells. In each histogram are indicated the proportion of positive cells and their mean fluorescence intensity. See M&M for the protocol. Data acquisition was performed on a FACSCalibur and analyzed with the CellQuest software.
